# Exploring the Potential of Endophytic Microorganisms and Nanoparticles for Enhanced Water Remediation

**DOI:** 10.3390/molecules29122858

**Published:** 2024-06-16

**Authors:** Madira Coutlyne Manganyi, Tshegofatso Bridget Dikobe, Mametsi Rahab Maseme

**Affiliations:** 1 Department of Biological and Environmental sciences, Sefako Makgatho Health Sciences University, P.O. Box 139, Medunsa 0204, South Africa; 2 Unit for Environmental Sciences and Management, Department of Botany, North-West University, Private Bag X2046, Mmabatho 2735, South Africa; 3 Department of Chemical and Physical Sciences, Walter Sisulu University, Private Bag XI, Mthatha 5117, South Africa

**Keywords:** endophytic microorganisms, nanoparticles, water remediation

## Abstract

Endophytic microorganisms contribute significantly to water bioremediation by enhancing pollutant degradation and supporting aquatic plant health and resilience by releasing bioactive compounds and enzymes. These microorganisms inhabit plant tissues without causing disease or any noticeable symptoms. Endophytes effectively aid in eliminating contaminants from water systems. Nanoparticles serve as potent enhancers in bioremediation processes, augmenting the efficiency of pollutant degradation by increasing surface area and bioavailability, thereby improving the efficacy and rate of remediation. Their controlled nutrient release and ability to stabilize endophytic colonization further contribute to the enhanced and sustainable elimination of contaminated environments. The synergistic effect of endophytes and nanoparticles in water remediation has been widely explored in recent studies, revealing compelling outcomes. Water pollution poses significant threats to human health, ecosystems, and economies; hence, the sixth global goal of the Sustainable Development Agenda 2030 of the United Nations aims to ensure the availability and sustainable management of water resources, recognizing their crucial importance for current and future generations. Conventional methods for addressing water pollution exhibit several limitations, including high costs, energy-intensive processes, the production of hazardous by-products, and insufficient effectiveness in mitigating emerging pollutants such as pharmaceuticals and microplastics. Noticeably, there is an inability to effectively remove various types of pollutants, thus resulting in incomplete purification cycles. Nanoparticle-enhanced water bioremediation offers an innovative, eco-friendly alternative for degrading contaminants. A growing body of research has shown that integrating endophytic microorganisms with nanoparticles for water bioremediation is a potent and viable alternative. This review examines the potential of using endophytic microorganisms and nanoparticles to enhance water remediation, exploring their combined effects and applications in water purification. The paper also provides an overview of synthetic methods for producing endophyte–nanoparticle composites to optimize their remediation capabilities in aqueous environments. The final section of the review highlights the constraints related to integrating endophytes with nanoparticles.

## 1. Introduction

Water is vital for life’s survival and maintaining the balance of ecosystems, serving as a fundamental resource for numerous biological processes and habitats. However, the acceleration of industrialization has resulted in increased water use, leading to the generation of industrial waste and the accumulation of contaminants in all areas of the ecosystem, including air, soil, and water, severely affecting the environment. These contaminants include heavy metals, chemical waste, and microplastics in water, as well as pesticides, herbicides, and persistent chemicals. Moreover, this has contributed to the emergence of water pollution as a prominent global environmental concern, especially for lower and middle-income nations [1]. The sixth global goal of the United Nations in the 2030 Sustainable Development Agenda aims to ensure the availability and sustainable management of water resources, recognizing their crucial importance for current and future generations [2]. However, conventional methods employed to tackle water pollution, such as chemical treatment and filtration, are energy-intensive, generate hazardous by-products, and offer inadequate efficacy in addressing emerging contaminants. Additionally, the inefficacy of alternative remediation approaches in removing various pollutants often leads to incomplete treatment and potential environmental risks [3]. Although these technological advancements have introduced more efficient purification techniques, these methods are hindered by their notably high cost, prompting a search for more sustainable solutions.

One such alternative approach involves the use of endophytic microbes. These beneficial microorganisms (such as bacteria and fungi) reside within plant tissues without causing harm. Endophytes offer promising solutions for bioremediation and environmental restoration and are adaptable to various environments [4]. These microorganisms can produce a wide range of enzymes and secondary metabolites capable of degrading or transforming various organic and inorganic pollutants, thereby improving the health of the environment [5]. The goal of bioremediation techniques is to transform organic substances into non-toxic forms in order to improve the health of the environment. Recent studies have highlighted the potential of fungi isolated from *Agrostis stolonifera* grasses, particularly when part of a consortium, to enhance the growth of *Festuca arundinacea grass* on lead-contaminated soil, suggesting their efficacy in heavy metal bioremediation [6]. Additionally, promising candidates such as *Aspergillus fumigatus*, *Rhizopus* sp., *Penicillium radicum*, and *Fusarium proliferatum* were identified among endophytic fungi for the remediation of hexavalent chromium in soil and the promotion of safer crop cultivation [7]. Additionally, studies on charophytes like *Chara subspinosa* have demonstrated their capacity to co-precipitate Cd, Cu, and Zn, providing a sustainable method for treating diluted mine drainage waters and reducing metal contamination [8].

In addition to endophytes, nanoparticles may serve as potent enhancers in bioremediation processes, increasing the efficiency of pollutant degradation by increasing surface area, stability, and bioavailability [9]. These enhancements ultimately improve the efficacy of remediation strategies. Nanomaterials are versatile, with dimensions in the typical range of 1 to 100 nm, and include metals, metal oxides, and polymers [10]. They have been used in various ways to treat contaminated water via a photocatalytic process [11]. Microbial nanoparticles such as silver, zinc, and titanium are known to possess properties that can inhibit the growth of viruses, bacteria, and other pathogens [12]. Integration of nanoparticles with filtration membranes has also shown promising outcomes [13]. Although numerous remediation approaches have been suggested, the utilization of nanoparticles as adsorbents is commonly recommended as a superior approach. For example, a silver nanoparticle and cellulose nanofiber composite prepared from *Citrus sinensis* showed adsorption chromium efficiency that exceeded 83% in the treatment of contaminated wastewater [14]. Zero-valent metals have been used as adsorbents for contaminants such as arsenic, lead, and cadmium [15]. The use of porous polymeric nanomaterials such as metal–organic frameworks (MOFs) as adsorbents has also received attention due to their additional structural flexibility, porosity, chemical stability, and reusability [16]. Magnetic nanoparticles, such as ferrites and multi-functionalized materials, have gained much attention due to their ease of recovery from remediated water and recyclability [17]. Microplastics (MPs) are considered to be a significant contributor to contamination in aquatic environments, including the ocean, sea, and river [18]. They pose a severe threat as they can act as carriers of pollutants, entering organisms and leading to bioconcentration and biomagnification along food chains [19,20]. This highlights the urgent need to address the detrimental impact of microplastic pollution on the environment and the interconnected food web. Shi et al. [21] demonstrated the effectiveness of using developed nanoparticles to remove microplastics from water using magnetic nano-Fe_3_O_4_. The average removal rate for common types of microplastics, including polyethylene, polypropylene, polystyrene, and polyethylene terephthalate, ranged from 62.83 to 86.87% based on type and size. This approach was effective for various environmental water bodies, achieving a removal rate of more than 80% and requiring minimal time [21].

The application of nanoparticles as sensors for detecting and monitoring water pollutants has also been explored [22]. Qasem et al. [23] and El Alouani et al. [24] highlighted the importance of developing and optimizing cost-effective and sustainable methods, paying special attention to various modern methods. A detailed comparative study of several conventional, advanced, and hybrid water treatment technologies was conducted [23]. In this study, the use of adsorbent-based materials was renowned as the most promising due to their simplicity, high metal removal rate, eco-friendliness, cost, and reusability. These methods include the use of carbon-, mineral-, biosorbent-, and MOF-based materials. Additionally, El Alouani et al. explored the use of thermally and mechanically stable geopolymers as adsorbents, which have shown capabilities of removing Pb, Cd, Cu, dyes, and organic compounds [24]. However, the lack of variability in the raw material composition and long-term stability remain challenges for upscaling.

**Figure 1:**
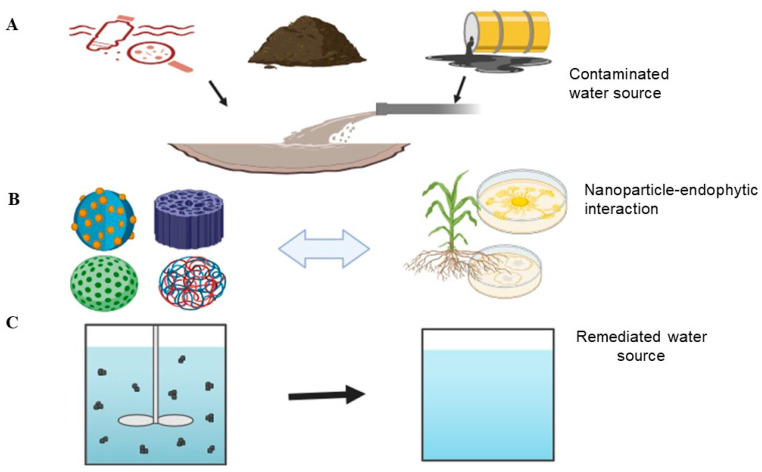
Schematic representation of water remediation using nanoparticles and endophytic microorganisms. (**A**) The process begins with contaminated water containing various pollutants (agricultural fertilizers, microplastics, and oil spills). (**B**) Various nanoparticles, such as iron oxide and titanium dioxide, are introduced to adsorb and neutralize contaminants in contaminated water. Simultaneously, endophytic microorganisms residing in plant roots degrade organic pollutants and enhance the efficiency of nanoparticles. (**C**) The combined interaction results in the removal of contaminants, yielding clean water. Arrows indicate the direction of interaction and remediation steps. Created with BioRender.com.

Given this background, the integration of endophytes and nanoparticles enables the exploration of creating synergistic systems that target and degrade contaminants in water more efficiently. In this approach, endophytes are employed in the biosynthesis of nanoparticles, including gold, silver, titanium, and magnetite nanoparticles [25]. Additionally, this approach offers potential solutions that are cost-effective, more sustainable, and environmentally friendly, as indicated in Figure 1. This review article outlines the individual use of endophytes and nanoparticles for water remediation and their combined effects. It highlights the biosynthesis process of nanoparticles and their application when integrated with endophytic microbiomes. It further provides an overview of nanoparticle synthetic methods and their potential for enhancing water treatment techniques, thus promoting environmental sustainability. The review presents a guide to address water pollution challenges through a biological and nanotechnology interdisciplinary approach, paving the way for viable alternative methods to improve water quality and environmental sustainability, as well as the constraints linked to integrating endophytes with nanoparticles.

## 2. Novel Developments in Endophytes and Nanoparticles in Water Remediation

The development of new nanomaterials for water clean-up has been the focus of recent research, especially when it comes to radionuclides and heavy metals [26,27]. The potential use of nanomaterials in this area has been highlighted by Guo et al. [26], who specifically discussed the efficiency of nanocarbon, mesoporous materials, and magnetic materials in the cleaning of various metals. Hernández-Hernández et al. [27] expanded in this field by exploring the use of biopolymer–clay nanocomposites as promising materials for pollutant removal, while Baigorria and Fraceto [28] emphasized the potential use of nanostructured materials. These studies underscore the potential significance of nanomaterials in water remediation, particularly in addressing the challenges posed by heavy metals, radionuclides, and organic contaminants.

In tandem with advances in nanomaterials, recent research has also explored innovative strategies for the use of endophytes and nanoparticles for water remediation. In particular, Ban et al. [29] and Polli et al. [30] studies investigate the collaborative use of endophytic bacteria and nanoparticles, showcasing synergistic effects in pollutant degradation and water purification. Ban et al. [29] emphasize the joint antimicrobial and catalytic activities achieved by combining endophytic bacteria with silver nanoparticles, underscoring the potential of such partnerships in multifaceted water treatment. On the other hand, Polli et al. [30] investigated the innovative use of endophytes in conjunction with magnetic nanoparticles, introducing magnetic responsiveness for facile separation after water treatment. These studies contribute to the growth of understanding endophytes’ and nanoparticles’ cooperative potential, providing new paths for the advancement of complex water treatment strategies.

## 3. Synthetic Methods of Endophyte–Nanoparticle Composites

**Figure 2:**
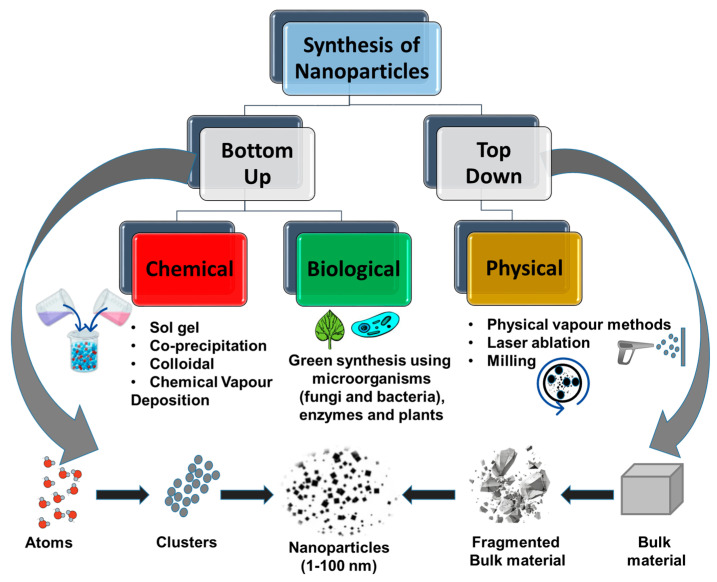
Classification of nanoparticle synthetic methods.

Nanoparticles can be synthesized using two main techniques: bottom–up chemical synthesis and top–down size reduction in bulk materials, as shown in Figure 2 [25,31,32]. Bottom–up synthesis involves the self-assembly of smaller building blocks (such as ions or atoms) that organize to form the desired nanoparticles. These nanoparticles can be synthesized through various chemical and biological methods. Chemical methods include sol–gel, co-precipitation, and chemical vapor deposition [33]. Deposition can occur from a liquid or a gaseous precursor. The chemical approach, however, often requires the use of toxic reagents and hazardous processes that are dangerous to the environment and human health. The spark discharge method has been employed as an eco-friendly alternative [34]. The method involves the passing of an inert carrier gas between two conductive electrode materials to produce an aerosol containing the ablated material that ultimately combines to form bigger particle clusters. These clusters agglomerate to form nanoparticles, which are consequently deposited on the desired substrate material. Biological entities such as microorganisms (usually fungi and bacteria), enzymes, and plants are used to mediate the conversion of ionic species into metallic nanoparticles [35]. The top–down synthetic methods, on the other hand, are destructive and involve a size reduction process of bulk materials or larger molecules through a mechanical or decomposition process, leading to the production of nanoparticles [25]. Sputtering, lithography, laser ablation, chemical milling, and biological size reduction are a few examples of this process [32,36]. In the biological size reduction approach, larger materials are biodegraded into smaller units to achieve the desired dimensions of 1–100 nm. The microorganism is incorporated into the process to influence the properties of the resulting nanoparticles or for their stabilization. An eco-friendly size reduction alternative method, such as the use of a hot injection-controlled aging technique, has been used [37]. The method involves the decomposition of nano-sized materials into smaller ones. In this method, an appropriate precursor is quickly injected into a hot surfactant-containing solvent, which leads to the sudden formation of nanocrystals. The method is beneficial for obtaining more precise control over nanoparticle size, shape, and morphology.

**Figure 3:**
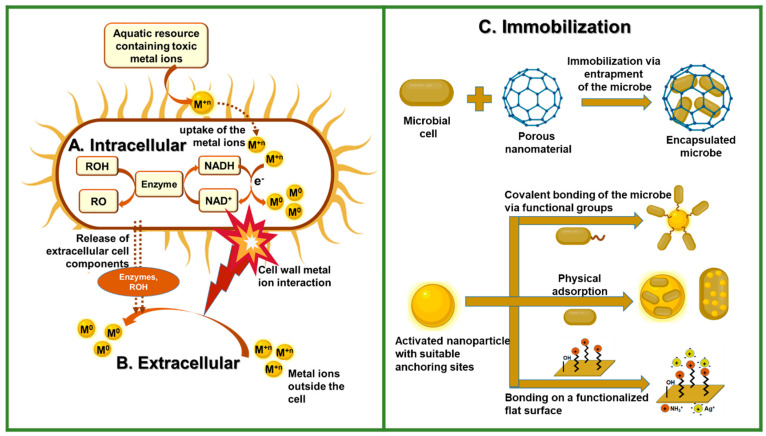
Schematic representation of the route for synthesizing biological nanoparticles. (**A**) Intracellular synthesis involves the transportation of ions into the microbial cell in the presence of enzymes and cofactors, which facilitate electron transfer reactions for the reduction of M^+^ to M^0^. (**B**) Extracellular synthesis occurs on the cell wall and in solution when the metal ions interact with extracellular cell components. (**C**) Immobilization of the microbe onto different surfaces via encapsulation, covalent bonding, and physical adsorption. Created with the assistance of BioRender.com.

Nanoparticles can be synthesized through biogenic, immobilization (or functionalization), and co-cultivation routes, as shown in Figure 3. When choosing a synthetic approach, it is important to take into account the properties of both the individual microbe and the nanoparticles, as well as the desired application.

### 3.1. Biogenic Synthetic Method

The biogenic synthetic method is often referred to as biomineralization or metal bioreduction. This synthetic method involves the use of the inherent biological processes of microorganisms such as bacteria and fungi to transform soluble metals, commonly known to be toxic, into insoluble, non-toxic zero-valent metal particles [25]. The ability of the method to induce the natural enzymatic and metabolic pathways of endophytes for the production of nanoparticles makes it an ideal, eco-friendly, and cost-effective alternative [38]. Endophytes have a high tolerance for toxic metals and thrive under severe environmental conditions. They contain functionalities and properties that can facilitate the reduction of metal species. For example, they produce a wide range of enzymes to catalyze the reduction process, and their oxidation–reduction potential and ability to produce secondary metabolites enable possible mediation in electron transfer pathways to facilitate the production of stable nanoparticles [39,40].

Their ability to secrete extracellular substances also contributes to the stability of the synthesized nanoparticles and the prevention of aggregate formation. Furthermore, endophytes can control the pH of their surroundings by secreting metabolites, which enhance the solubility and kinetic behavior of the metal species [41]. All things considered, these characteristics enable them to endure in hazardous surroundings, which either directly or indirectly contribute to their reduction potential. Silver nanoparticles synthesized by treating silver nitrate with plant extracts from *Plantago ovata* [39] and *Viburnum opulus* [40] were shown to be highly effective in degrading harmful organic dyes. Metabolites responsible for the reduction and efficient stabilization of the nanoparticles were identified as polyphenols, phenolic acids, and flavonoids [40]. Nanoparticles synthesized via biological methods tend to have smaller particle sizes, hence a higher specific surface area and superior activity [39,40,42].

The metal reduction process can be intracellular or extracellular [43,44], as shown in Figure 3A,B. Intracellular synthesis methods involve the formation of nanoparticles inside the microbe cells via the transportation of ions and molecules into the cells, as facilitated by either enzymes or other bioactive molecules. Through electrostatic attraction, functional groups on the cell wall, such as carboxyl groups, interact with the metal ions. This is followed by the reduction of the metal ion to its metallic form. The method depends on the employed microorganism’s metabolism once the cell takes up metal ions and often requires additional steps that include sonication to disrupt the microbial cell wall and purify the nanoparticles through multiple extractions [45]. On the contrary, the extracellular method involves the entrapment of metal ions on the exterior of the bacterial or fungal cell walls, thereby allowing the formation and stabilization of the nanoparticles to occur externally with the assistance of enzymes and metabolite secretion [46].

Research on the production of various metal nanoparticles both inside and outside of cells, as well as their antibacterial qualities in water treatment, has been published [25,47]. For example, gold nanoparticles were synthesized extracellularly and intracellularly via a bio-reduction process facilitated by the algae *C. sorokiniana* [43]. The extracellular method was achieved using the extracts, while the intracellular method was achieved using growing cells. Intracellular particles were larger (20–40 nm) than those produced extracellularly (5–15 nm). However, in another study, the opposite was observed [48]. Herein, extracellular nanoparticles with 91 nm particle size were more stable and efficient in reducing methylene blue (MB), with a twenty-two times faster reduction rate compared to the intracellular ones (45 nm). Data analysis suggested that the nanoparticles were formed due to the attachment of metallic ions to the electron-donating bioorganic compound, which promotes their stability. Furthermore, the nanoparticles synthesized internally accumulated on the exterior wall, causing bacterial cell wall damage [48]. Factors such as precursor salt concentration, pH, and reaction time played a role in determining the properties of the nanoparticle produced with regard to agglomeration, stability, and activity. TEM images revealed that significant cell damage had taken place following treatment with the HAuCl_4_ solution. In another study, the interaction between toxic Au (III) soluble species and the *Delftia acidovorans* bacterium grown on agar plates was reported to generate the formation of a secondary metabolite, which promoted the biomineralization of gold, serving a protective role against toxic soluble gold [49]. Furthermore, dead biomass from the fungus *Hypocrea lixii* was used to promote the uptake of nickel ions for the production of nickel oxide nanoparticles. This marked a significant advancement in nanobiotechnology since the generation of nanoscale metal oxides is relatively unknown. Nanoparticles were accumulated inside the cell and on the external cell-wall surface [50].

When Au, Ag, and bimetallic Au–Ag nanoparticles were synthesized using leaf broth, it was suggested that the reduction process to form the nanoparticle could be facilitated by the extracellular sugars or terpenoids, which may stabilize the nanoparticle [51,52].

### 3.2. Synthesis by Immobilization

Immobilization of microorganisms can occur through encapsulation, physical adsorption, and bonding of the microbe to the nanoparticle surface through covalent bonding, as shown in Figure 3C. The method aims to fix or confine the microorganisms or their active components onto a nanomaterial support [53,54]. Using immobilized microorganisms instead of free ones offers several advantages. They are easier to separate from the product; they experience increased productivity due to a higher surface area; and the protection of the microorganisms against harsh environmental conditions is further enhanced. This promotes their stability, which further improves their reusability, consequently leading to lower input industrial costs [55].

Bacteria immobilized on nanoparticles can be used to remove environmental pollutants, acting as efficient bioremediation agents. The integration of immobilization technologies not only provides adsorbents with improved mechanical strength, porosity, and increased adsorption capacity but also improves their resistance to environmental changes. For example, water treatment methods containing immobilized endophytic cells have demonstrated a higher removal efficiency of ciprofloxacin (CIP) antibiotics compared to those with free cells [56,57]. This performance was attributed to the efficient multiplication of the bacterial cells on the support material in contrast to the suspension form, resulting in greater degradation of CIP and other organic contaminants.

Endophytes can be encapsulated in nanomaterials to allow their confinement within nanoscale matrix structures [58]. The encapsulation process can enhance endophyte stability, viability, and controlled release, making them more effective for various applications [59]. For example, microorganisms encapsulated within hydrogels were shown to improve the microorganism’s tolerance against unfavorable environments [60,61,62]. In another study, endophytic activity was increased ten-fold when the bacteria *Kosakonia radicincitans* was encapsulated into a biopolymeric material [63].

Bacterial cells can also adhere to the surface of a nanoparticle through weak physical interaction (such as van der Waals). The process requires minimum energy, is flexible, and, most importantly, the adsorbent can be reused continuously, proving to be extremely cost-effective. Strong covalent chemical bonds can be formed between the functional groups of the fungal or bacterial surface and the surface of the nanoparticle [64]. This may occur via a condensation reaction with a release of water or alcohol, depending on the nature of the molecules involved in the reaction. Endophytic organisms possess complex cellular structures with main functional groups found in the structures that include functionalities containing hydroxyls and carbonyls such as carboxyl (-COOH), ketones, aldehydes, esters, and phenols, as well as amino groups (-NH_2_), phosphoryl, and thio (-SH) groups [65]. These functional groups diversify endophytes and contribute to their overall polarity, stability, and reactivity. On the other hand, nanoparticles typically contain surface hydroxyl, hydride, and chloride groups that strongly bind via the endophytic functional groups with simultaneous loss of either water, salt, or alcohol, depending on the functional groups involved. González-Ballesteros et al. [66] highlighted the significance of the total phenolic content in marine *Cystoseira baccata* algae for green synthesis. Venkatesan et al. [67] demonstrated the efficacy of alcohols and hydroxyl functional groups in *Ecklonia cava* extract. For example, strains of *Acinetobacter lwoffii* ACRH76, *Bacillus pumulis* C2A1, and *Acinetobacter* sp. HN_3_ were immobilized on polystyrene sheets and showed improved CIP remediation compared to when free strains were used [68]. Furthermore, characterization conducted before and after the synthesis of the nano-biocomposite revealed the effectiveness of alkenes, carbonyl contents, proteins, and notably aromatic compounds associated with the extract of *C. sorokiniana* [43]. The success of interactions between endophytes and nanoparticle interactions can be monitored with the Fourier transform infrared (FTIR) technique, which tracks the disappearance and generation of chemical bonds during a chemical reaction [69,70]. For example, the reduction in the intensity of the OH absorption band in the fungus–Fe_3_O_4_ composite after conjugation suggested that bonding occurred through the Fe_3_O_4_ hydroxyl surface groups. The technique was also used to identify the functional groups of bioactive molecules responsible for reducing Au ions and stabilizing luminescent Au_2_S nanoparticles derived from *Humicola* sp. [71]. Additionally, FTIR was utilized during the reduction of Ag(I) to Ag(0) by the marine fungus *Cladosporium halotolerans* [72] and during the *Penicillium brasilianum* NP5-mediated synthesis of AgNPs [73].

### 3.3. Synthesis by Co-Cultivation

Synthesis by co-cultivation involves the combination of the endophyte and pre-synthesized nanoparticles in a culture medium, allowing interactions to occur. It is a commonly used synthetic technique for bionanomaterials because of its simplicity and the fact that it does not require extensive work. The method involves the mixing of an endophyte extract and a metal precursor in the presence of a stabilizing agent. Careful control of reaction conditions is crucial to obtaining nanoparticles that are application-specific. These conditions include pH, reaction, medium, and temperature; the type of stabilizing agent used; and the mixing method—for example, normal stirring, ultra-sonication, or shaking. The type of shaking method used dictates the uniformity of the precursor and ultimately influences the physical properties of the nanoparticle and, hence, its application. For example, a magnetite nanoparticle (Fe_3_O_4_)—*Aspergillus flavus* nanobiocomposite (*Af*-CL-7) showed exceptional bioremediation capacity and effectiveness in reducing the toxicity of reactive black 5 (RB5) textile dye while enabling its regeneration and reusability [30]. The nanocomposite was typically synthesized by reacting a solution of magnetite with a medium containing the microorganism. The interaction between these two components may occur in various forms. Kumar et al. [74] investigated the synthesis of silver nanoparticles using a culture supernatant of the strain *Pseudomonas aeruginosa.* Analysis by FT-IR indicated that the nitrate reductase and the rhamnolipids produced during cultivation might be responsible for the reduction and capping of the crystalline nanoparticle. Additionally, biologically pre-synthesized silver nanoparticles were further layered with a biodegradable chitosan matrix to enhance the properties of the bionanocomposite system [75]. As a result of this fabrication, stabilizing exopolysaccharides were produced and shown to provide extended stability and prevent agglomeration.

## 4. The Effect of Combining Endophytes and Nanoparticles for Water Remediation

The synergistic effect of endophytes and nanoparticles in water remediation has been widely explored in recent studies, revealing compelling results. He et al. [76] and Kamyab et al. [77] highlight the potential of nanomaterials in water treatment, with the former emphasizing their use in polymer composites and the latter focusing on their catalytic and antimicrobial properties. Bhandari and Shukla [78] further demonstrated, respectively, the efficacy of biomass-derived nanocomposites in pollutant removal and disinfection and the use of iron-based nanoparticles as adsorbents in water purification.

In a study by Elbahnasawy et al. [79], the researcher investigated the improved remediation capabilities achieved by combining *Rothia endophytica* with silver nanoparticles, focusing on their joint antimicrobial and catalytic activities. Similarly, the work of Sathiyaseelan et al. [80] delves into the use of endophytic fungi in conjunction with titanium dioxide nanoparticles for the degradation of organic pollutants in water, highlighting the potential for advanced photocatalytic processes. A study conducted by Tabande et al. [81] extends the exploration of nanomaterials in water treatment by emphasizing the multifunctionality of zinc oxide nanoparticles combined with endophytes, showcasing their ability to adsorb and degrade contaminants simultaneously. In a complementary manner, Hao et al. [82] investigated the application of carbon-based nanoparticles in collaboration with endophytic bacteria for the removal of heavy metals from water, shedding light on their promising adsorption capacities.

The research carried out by Liosis et al. [83] explores the innovative use of endophytes in combination with magnetic nanoparticles for efficient water purification. Their findings emphasize the magnetic responsiveness of the nanoparticles, facilitating easy separation after the water treatment processes. Additionally, the study by Sur [84] investigated the potential of graphene-based nanoparticles in synergy with endophytic microorganisms, showcasing their prowess in pollutant adsorption and subsequent removal from water matrices.

The work of Ali et al. [85] contributes to the body of knowledge by examining the collaborative impact of endophytic bacteria and gold nanoparticles, emphasizing their joint capabilities in pollutant degradation and heavy metal removal. Similarly, the study by Wu et al. [86] explores the use of copper-based nanoparticles in conjunction with endophytes to remove antibiotic residues from water, demonstrating their effectiveness in the treatment of emerging contaminants.

The research carried out by Kaabo [87] focuses on the combination of *Penicillium oxalicum* and silica nanoparticles, demonstrating their potential in the sequestration of organic pollutants and heavy metals. Furthermore, the study by Gran et al. [88] investigates the use of cerium oxide nanoparticles along with endophytes for the simultaneous removal of organic dyes and heavy metals, highlighting the synergistic effects in water remediation. These studies collectively strengthen the evidence supporting the effectiveness of combining endophytes and nanoparticles for water remediation. The diverse applications of various nanoparticles, in collaboration with different endophytic microorganisms, offer a promising avenue for developing advanced and efficient water treatment strategies.

Bioremediation is a biological means of recycling environmental waste into a variety of important products that can benefit other organisms. This method of biological pollution treatment is highly sustainable and affordable and follows safe remediation techniques [89,90,91]. Endophytes inhabit the internal organs of their plant hosts during a specific period of their lives. Endophytic microorganisms are diverse groups with an estimated one million species [92,93,94]. The literature states that billions of live plant species in natural ecosystems are hosts for various endophytic microbes [95]. Thus, this group of microbes is one of the most significant untapped natural resources for the bioprospecting of biosynthetic enzymes and secondary metabolites [95].

In addition, endophytes are preferred to plants in remediation due to their ease of growth, rapid growth period, and easy manipulation [96,97]. Environmental polluters may come from varying sources, such as mining areas, water runoff (dams and sewage, etc.), sediments, agricultural land, and heavily polluting industries [98]. Various biological agents are used for bioremediation to clean up contaminated sites. Bacteria, archaea, and fungi are common bioremediators used [99]. Notably, bioremediation serves as a hotly debated research topic due to the importance of microorganisms in addressing environmental contamination threats. Numerous metal contaminants are produced by various industries that affect the soil and aquatic environments. For example, pollutants from the dye industry produce different heavy metals that are toxic to the environment [100]. In addition, untreated pollutants from wastewater from the agri-food industries disposed in rivers and other water bodies seriously affect the environment [101]. The heavy metals produced are highly toxic to aquatic and terrestrial habitats and their inhabitants. For example, in humans, mercury, cadmium, and lead alter the central nervous system, especially in infants, while lead causes liver and kidney dysfunction, cardiovascular disease, and malfunction of the reproductive and immune systems [102,103]. With all these negative effects of heavy metals, pesticides, herbicides, organic chemicals, and other substances on the aquatic environment posing a huge risk to the well-being of humans, animals, and plants, this calls for an intervention.

The green synthesis of nanoparticles (NPs) seems to be a sustainable solution for the removal of water pollutants. Specifically, the use of endophytic microorganisms to synthesize these nanoparticles is a more beneficial method compared to other physical and chemical methods. They are also eco-friendly, cost-effective, and produce nanoparticles with significant morphology and size. It has been reported that microbe-mediated NP synthesis demonstrated an upper hand over NP biosynthesis in algae and plants [104]. Endophytic NP synthesis methods have gained more attention in various fields, including medical, pharmaceutical, environmental, and agronomical applications. In addition, fungi are considered a better choice for nanoparticle synthesis, as they secrete abundant enzymes and secondary metabolites and act as easy targets for growth [44]. Most endophytic microorganisms such as bacteria, fungi, and actinomycetes tend to convert metal ions into metallic NPs such as Ag, Au, Zn, and Cu through the secretion of secondary metabolites and cellular enzymes [105].

Several bacteria, such as *Pseudomonas aeruginosa*, *Bacillus subtilis*, *Rhizobium gallium*, and *Staphylococcus aureus,* were used for their high adsorptive potential for the bioremediation of toxic metal ions in wastewater [106]. Verma et al. [107] reported the use of sulfate-reducing bacteria (SRB) to treat wastewater containing chromium. In one study, copper nanoparticles were synthesized from *Escherichia* sp. SINT7, which exhibited copper resistance [108].

Carbon-based materials, such as nanocomposites and nanotubes, and metal oxide-based materials, have been used for effluent removal from pharmaceutical wastewater through methods such as photocatalytic degradation, adsorption, and nanofiltration [109]. Kariim et al. [110] used multiwalled carbon nanotubes (MWCNTs) from Fe–Ni, which were used to remediate metronidazole and levofloxacin from pharmaceutical wastewater. Similarly, the efficiency of metal oxide-based nanomaterials in effluent treatment was noted when nanoporous magnesium oxide from solid waste from the ductile iron industry adsorbed 1000 mg·g^−1^ of toxic dye from wastewater [111]. Mahanty et al. [112] developed iron oxide nanoparticles using *A. tubingensis* (STSP 25) obtained from the rhizosphere of *Avicennia officinalis* in the Sundarbans, India. These nanoparticles were highly effective in removing heavy metals such as Pb (II), Ni (II), Cu (II), and Zn (II) from wastewater. Iron–sulfur nanoparticles were also used to break down Naphthol Green B dye by transferring electrons extracellularly, inhibiting the production of harmful gases and metal complexes [113].

Another approach was through technology that can convert biological waste into useful products for water bioremediation, creating a two-way winning strategy that reduces waste and generates useful products simultaneously. This strategy converts waste using microorganisms and nanotechnology to produce products such as adsorbents, clinker, biogas, biohydrogen, and biomolecules [114]. For example, nanoparticles were used to enhance dark fermentation reactions to increase biohydrogen production [115]. Another way is to supplement fermentative bacteria with nanoparticles, where Elreedy et al. [116] used mixed culture bacteria in conjunction with single, dual, and multiple nanoparticles to generate biohydrogen. Adding nickel oxide and hematite nanoparticles resulted in 1.2–4.5 times greater biohydrogen production compared to nanoparticles alone.

Various fungal species, such as *Aureobasidium pullulans*, *Aspergillus niger*, *Cladosporium resinae*, *Penicillium* species, *Funalia trogii*, *Ganoderma lucidum*, *Rhizopus arrhizus*, and *Trametes versicolor*, effectively absorbed heavy metals from polluted sites and produced nanoparticles [117]. An accumulating body of evidence supporting the combination of endophytes and nanoparticles for water remediation is summarized in Table 1. These studies provide valuable information on the diverse applications and innovative combinations of nanomaterials, strengthening the potential for advanced and efficient water treatment strategies. Salvadori et al. [50] found that the dead biomass of *H. lixii* could absorb Cu (II) and produce copper nanoparticles. Furthermore, *H. lixii* produced extra- and intra-cellular NiO nanoparticles [50,118].

## 5. Application of Endophytes and Nanoparticles in Water Remediation

The promising results of the use of endophytes and nanoparticles in water remediation have encouraged extensive research on their applications and effectiveness [54,129]. Nanomaterials, such as titania, carbon nanotubes, zero-valent iron, and silver, have been effective in purifying water through various mechanisms, including the adsorption of heavy metals and other pollutants, the removal and inactivation of pathogens, and the transformation of toxic materials into less harmful compounds [130,131]. These materials have also been used in the remediation of soil and groundwater, particularly for heavy metals, inorganic and organic contaminants, and emerging contaminants [132]. In the realm of groundwater remediation, specific types of nanomaterial have emerged as particularly promising. For example, carbon-based nanomaterial sorbents and zero-valent iron nanoparticles have demonstrated significant potential in the removal of organic and inorganic contaminants from groundwater [133]. Their ability to effectively target a wide range of contaminants makes them versatile tools for the pursuit of clean and safe water resources. The high surface area and reactivity of these nanomaterials make them more efficient in environmental remediation than conventional techniques, marking a significant advancement in the field [134].Recent studies have delved deeper into the specific applications and mechanisms underlying the success of nanomaterials in water remediation. The multifunctional properties of graphene-based nanoparticles were explored, demonstrating their effectiveness in the simultaneous adsorption and degradation of contaminants in water matrices [84,106]. Additionally, the potential of combining nanoparticles with endophytic microorganisms showcases a synergistic approach for improved water purification [129]. Selected studies that focused on combining endophytes with nanoparticles for water remediation are summarized in Table 1. For example, silver nanoparticles synthesized by *Bacillus* spp. served as adsorption sites for heavy metal contaminants, including cadmium, chromium, nickel, and copper, thereby enhancing the microbial reduction of these ionic metals to their elemental state [119]. Furthermore, they assist in the phytoremediation and stimulation of plant growth, which can be applied in the agricultural sector [119]. The bacteria from the genus *Bacillus* utilize uptake, transformation, and detoxification methods to decontaminate water from heavy metals. More studies have demonstrated that these methods are effective in removing CIP from pharmaceutical wastewater, offering potential benefits to the medical healthcare industry [68,123]. Biogenic palladium nanoparticles were synthesized through the adsorption and reduction of Pd(II) ions on the surface of wastewater from *Escherichia coli*. These nanoparticles were further utilized to selectively catalyze the reductive degradation of CIP in the presence of hydrogen gas. The ability to recover and utilize palladium contributes to the pursuit of a cleaner ecological environment. Additionally, the demonstrated scale-up potential offers an efficient method for CIP removal. The efficiency of the method encourages further technological developments for industrial applications [123]. Immobilized *E. coli* was shown to enhance the removal of CIP in floating treatment wetlands (FTWs). The FTWs utilize a combination of plants and bacteria to degrade and remove pollutants. The immobilized system provides a surface area for enhanced bacterial growth and multiplication, resulting in biofilm formation. FTW methods have become popular due to their cost-effectiveness in removing contaminants from water [68]. The potential application of endophytic nanoparticles was further demonstrated when the gold-resident bacterium *Delftia acidovorans* facilitated the biochemical gold biomineralization process through the production of secondary metabolites. These results indicate great potential for reducing the environmental impacts caused by conventional gold extraction methods [121]. Functionalized magnetic iron oxide was effectively used to adsorb copper in dyes from industrial effluents [122]. The recovery of silver was facilitated by manganese oxide produced by a deep-sea bacteria *Marinobacter* sp. MnI-79 [124]. 

## 6. Limitations and Risks of Using Endophytes and Nanoparticles in Water Remediation

Although the capabilities of endophytes in the degradation of organic pollutants and the reduction of toxic ionic metal species are remarkable, it is important to recognize and address the associated limitations. As these advancements move from laboratory experiments to real-world applications, distribution, practicality, and economic viability become critical considerations. The slow growth of endophytes presents practical challenges to achieving rapid and extensive degradation of water pollutants. These constraints pose a hindrance to their effectiveness in large-scale pollutant remediation and, consequently, their commercialization. Furthermore, deploying endophytic nanoparticles in large-scale water treatment processes is limited by the cost associated with synthesizing and storing them. The use of nanoparticles may possibly introduce new environmental and human health hazards [135]. Martinez et al. [136] investigated the long-term stability and environmental adaptability of endophyte–nanoparticle complexes. Ensuring the effectiveness of these complexes over extended periods and under varying environmental conditions is crucial for sustained water remediation efforts. Furthermore, there is concern regarding the use of silver nanoparticles in consumer products due to their potential release into the environment [137]. Studies have shown that these nanoparticles can accumulate in aquatic animals and eventually make their way into the food chain, posing a potential risk to human health. Although surface modifications can increase their stability against aggregation and deposition, it is still important to consider their removal from water and wastewater to prevent their accumulation in the environment [138]. Addressing these concerns is imperative to ensure responsible and sustainable implementation of nanomaterials in water treatment strategies.

It is evident, therefore, that the complex interactions between nanoparticles, living organisms, and ecosystems require careful consideration to avoid unintended consequences. Understanding the fate and transport of nanoparticles in water systems is crucial for anticipating their behavior and potential consequences for ecosystems. For example, the microbial communities that already exist in water bodies can be severely disrupted by the introduction of foreign microorganisms or engineered microbes for remediation purposes. Because of the potential for detrimental ecological imbalances and the loss of native biodiversity, this disturbance is extremely concerning. To mitigate unintended consequences, it is essential to have a comprehensive understanding of how nanoparticles behave in water systems. Various studies have reported the consequences and hazardous impact of heavy metals and metalloids that could be treated with biogenic nanoparticles [139,140,141,142]. Nanoparticles can undergo bioaccumulation, biotransformation, and magnification. Bioaccumulation refers to the uptake of contaminants and the subsequent increase in their concentration in aquatic organisms. For example, Dube and Okuthe [139] conducted a detailed study on the movement and mechanistic transformation of engineered nanoparticles within aquatic environments, with a focus on their toxicity in fish. The biomagnification of silver nanoparticles was demonstrated to elevate trophic toxins [140]. Additionally, the accumulative effect of biomagnified TiO_2_ inhibited the growth of algae in the aquatic food chain [141]. Native species frequently lose out to introduced or genetically modified microbes for resources like nutrients and available space, which reduces native population levels and modifies the structure of the community [142]. The overall health of the ecosystem may be impacted by these alterations to important symbiotic relationships, such as those between microbes and aquatic plants or animals. Furthermore, the introduction of microorganisms can lead to selective pressures that favor some species over others, further upsetting the natural balance of microbes and hindering vital ecological processes like the cycling of nutrients and the breakdown of organic matter [143]. Preserving beneficial organisms and preventing adverse effects on ecosystems are critical factors to consider during the development and application of these technologies. Compliance with existing regulations and the establishment of ethical guidelines are vital to ensuring the responsible application of these technologies. Ethical considerations, public perception, and regulatory frameworks play crucial roles in shaping the future of these remediation strategies.

Having emphasized the need to understand the navigation and the potential environmental and health impact of nanomaterials, it is evident that the pace at which innovations are introduced far exceeds the rate at which scientists may develop a comprehensive understanding of their impact. As more sophisticated nanoproducts are generated, they present changes in aquatic systems due to their properties and functionalities, making it extremely difficult to keep up. To effectively mitigate the risks highlighted in this review, it is fundamental to conduct a thorough environmental impact assessment and develop application strategies that can be controlled and monitored to limit unexpected consequences. This is an assessment tool that aims to identify the environmental and socio-economic impacts of water treatment interventions before their use [144,145]. Environmental impacts can be predicted during the early design and development stages of new bioremediation strategies. In this way, the impact of adverse effects can be predicted and consequently reduced by opting for less destructive remediation strategies. Assessment guidelines would, therefore, need to be specific to the type of nanomaterial since their behaviors cannot be extrapolated. Risk management for nanomaterials can be investigated in a standardized laboratory setting; however, the experimental conditions may not be representative of natural conditions that are often affected by various artifacts. In general, the steps followed to design and improve the risk assessment of nanomaterials first involve prioritizing the identification of the nanomaterials that are predicted to express higher reactivity and toxicity. This is followed by toxicity measurements for all the identified materials, followed by the development of equipment that can measure the nanomaterials in environmental samples to define their concentrations in aquatic ecosystems. Finally, toxicity tests can be conducted on well-defined nanoparticles under standardized conditions and predict their fate through tests on environmental and laboratory samples. For example, Ramirez et al. [145] used the fuzzy logic methodology to assess the risk of silver nanoparticles in two aquatic system cases: an effluent wastewater plant and an accidental spill. The method dealt with variables associated with toxicity uncertainties, such as shape, size, and coating. According to the results, pollutants released from accidental spills had a higher risk compared to wastewater plant releases based on their concentrations. The approach is considered flexible and comprehensive for application in different scenarios [146,147,148]. Through computational models, pollutant concentrations in aquatic systems can be estimated [148,149] and continuously monitored.

## 7. Conclusions

Integrating endophytic microorganisms with nanoparticles for water bioremediation is a novel approach that shows promise in addressing water pollution issues. Incorporation of endophytic microorganisms with nanoparticles can provide several benefits in water bioremediation, including increased microorganism stability, dispersibility in aqueous environments, increased pollutant degradation capabilities, and improved resistance to environmental stressors. Furthermore, nanoparticles can transport endophytic microorganisms to specific contamination sites, increasing their accessibility and activity in polluted water. Combining endophytic microorganisms and nanoparticles is a promising strategy for developing long-term and efficient water bioremediation solutions. Despite the potential benefits of using endophytes and nanoparticles in water remediation, it is essential to acknowledge and address the associated limitations. Notably, their slow growth rate is a hindrance to upscaling and commercial distribution. Therefore, research and development are necessary in this area to improve the performance of these hybrid systems and promote their widespread use in addressing water pollution issues. The long-term negative environmental and ecological effects cannot be ignored, as indicated by several studies. However, these can be combated by regularly conducting comprehensive environmental impact assessments and computational models to identify potential hazards early on and monitor them over time.

## Figures and Tables

**Table 1:** Selected studies on the Combination of Endophytes and Nanoparticles for Water Remediation.

Endophytic Microorganism	Pollutant Remediated	Nanoparticle Type	References
*Bacillus cereus*	Methylene Blue dye	Silver	Alfryyan et al. [48]
*Aspergillus tubingensis* STSP 25	Removal of Pb (II), Ni (II), Cu (II), Zn(II)	Iron oxide	Mahanty et al. [112]
*Bacillus* spp.	Cadmium, chromium, nickel, and copper	Silver Nanoparticles	Wróbel et al. [119]
*Pseudomonas aeruginosa* JP-11	Removal of Cadmium, chromium, uranium, nickel and copper.	Cadmium Sulphide	Chellaiah et al. [120]
*Delftia acidovorans*	Toxic gold ions.	Gold	Johnston et al. [121]
*Streptomyces thermolineatus*	Removal of copper	Iron oxide magnetic	Suganthi S. et al. [122]
*Escherichia coli*	Removal of ciprofloxacin from hospital wastewater.	Biogenic Palladium	He et al. [123], Shah et al. [68]
*Marinobacter* sp. MnI-79	Removal of Ag^+^	Manganese oxide	Pei et al. [124]
*Penicillium*	Textile wastewater (methylene)	Iron	Mathur et al. [125]
*Hypocrea lixii*	Heavy metal mine.	Copper	Salvadori et al. [50,118]
*Penicillium* sp.	Deep sea hydrocarbon oil reserves.	Nickel oxide	Velez et al. [126]
Manglicolous fungi	Removal of Cr (VI)	Iron oxide	Mahanty et al. [127]
*Scenedesmus incrassatulus*	Removal of chromium (VI), cadmium (II)	Palladium	Pena-Castro et al. [128]

## Data Availability

Not applicable.
